# Coaching: a new model for academic and career achievement

**DOI:** 10.3402/meo.v21.33480

**Published:** 2016-12-01

**Authors:** Nicole M. Deiorio, Patricia A. Carney, Leslie E. Kahl, Erin M. Bonura, Amy Miller Juve

**Affiliations:** 1 Department of Emergency Medicine, Oregon Health & Science University, Portland, OR, USA; 2 Department of Family Medicine, Oregon Health & Science University, Portland, OR, USA; 3 Department of Medicine, Oregon Health & Science University, Portland, OR, USA; 4 Department of Anesthesiology and Perioperative Medicine, Oregon Health & Science University, Portland, OR, USA

**Keywords:** formative feedback, coaching, education, medical, undergraduate, psychometrics, counseling, mentors, medical education, faculty development

## Abstract

**Background:**

Individualized education is emerging as an innovative model for physician training. This requires faculty coaching to guide learners’ achievements in academic performance, competency development, and career progression. In addition, coaching can foster self-reflection and self-monitoring using a data-guided approach to support lifelong learning.

**Context:**

Coaching differs from mentoring or advising, and its application in medical education is novel. Because of this, definitions of the concept and the constructs of coaching as applied to medical education are needed to accurately assess the coaching relationship and coaching processes. These can then be linked to learner outcomes to inform how coaching serves as a modifier of academic and competency achievement and career satisfaction.

**Innovation:**

We developed definitions and constructs for academic coaching in medical education based on review of existing education and non-education coaching literature. These constructs focus on 1) establishing relationship principles, 2) conducting learner assessments, 3) developing and implementing an action plan, and 4) assessing results and revising plans accordingly.

**Implication:**

Coaching is emerging as an important construct in the context of medical education. This article lays the vital groundwork needed for evaluation of coaching programs aimed at producing outstanding physicians.

Individualized, competency-based, learner-driven education is emerging as an innovative model for physician training (1). Support for this model includes Knowles’ framework of six principles of adult learning, which postulates that adults are internally motivated and self-directed, bring life experiences and knowledge to learning experiences, are typically goal and relevancy oriented, are practical, and like to be respected (2). Some medical schools are now undertaking individualized, student-driven approaches to training medical students. There is much to be learned about how to ensure the success of this approach (1).

For individualized training to produce competent physicians, faculty must foster students’ lifelong learning skills, especially for those at the very beginning of their training. While mentoring and career advising are typically provided during undergraduate and graduate medical education, addressing learners’ deficits usually occurs only when learners encounter academic difficulties. Fostering lifelong self-monitoring skills should start at entry into medical school and should focus on increasing students’ abilities to 1) set learning goals that mitigate gaps in knowledge and skills, 2) seek opportunities to meet these goals, and 3) determine their readiness for advancement. Coaching offers the opportunity to improve self-monitoring by fostering habits that will identify deficits and create goals to mitigate them early in the educational process.

## Context

The constructs of coaching provide a better fit than mentoring or advising for individualized, learner-driven education. Coaching has been described in the business literature (3) and, more recently, in higher education publications. However, it has only started to emerge in the medical education literature (4–7). The model of continuous professional development needed to stay up-to-date in medicine will require advancing these learner-driven skills in medical students, and coaching can be a tool to develop these abilities (8).

Since coaching as applied to medical education is novel, we need to define what coaching relationships and coaching processes look like, and how these differ from mentoring or advising. Developing these constructs will assist in identifying precise measures to determine the effectiveness of coaching and how the relationship and processes used interact to produce highly competent physicians. The purposes of this article are to 1) define the concept of coaching and create a conceptual framework applied to medical education and 2) identify and define constructs for measurement.

## Innovation

### Coaching as applied to medical education: creating a conceptual framework

In 2015–2016, we conducted a literature review on coaching across many domains. We found that literature on coaching in medical education is limited (4–7). We then searched domains in health professions and traditional higher education, sports, and business to identify applicable literature. Finally, we reviewed relevant literature on mentoring and advising in undergraduate medical education (9).

We found the business literature's analyses of executive coaching provided the best application to ongoing coaching of medical learners (3). While mentoring is similar to coaching in that it involves establishing a relationship between a more experienced individual and a less experienced individual, mentoring is more directive, with the mentor intentionally transferring specific knowledge and skills and guiding the learner's activities (3). Similarly, advising is described as a faculty-dominated process where a learner comes to a faculty member with specific questions and is given direction regarding his or her academic, social or personal matters. The constructs that underlie advising and mentoring contrast distinctly with coaching, which is learner-driven (9).

Often, advisors and mentors are only provided with filtered performance information provided from their learners, and they may not be privy to all relevant information about a student. Unlike mentors or advisors, coaches do not offer advice or therapy to learners they are coaching. Rather, after reviewing objective data on performance, coaches employ methods that help the learners gain insights into their own assumptions, clarify meaning about relevant outcomes, and help identify specific actions needed to achieve a desired result (3). Such methods include asking reflective questions to help learners identify and develop personal values, preferences, and unique perspectives. These approaches can stimulate lifelong master adaptive learning skills (8).

With graduate medical education now focused on milestone achievement, and undergraduate medical education increasingly focused on competency development, coaching can help learners reflect on where their performance stands and how to improve. Eva and Regehr indicate that a learner's ability to self-monitor *via* self-reflection, without input from others, is suboptimal (10, 11). These authors further posit that a failure exits in properly explicating the role of self-assessment in the self-regulating profession of medicine. Coaching can help develop skills that will empower students to become more reflective in developing self-monitoring and lifelong learning skills and to reach their full potential.

To summarize, *the goal of coaching as applied in medical education* is to support a developmental process whereby an individual learner meets regularly over time with a faculty coach to create goals, identify strategies to manage existing and potential challenges, improve academic performance, and further professional identity development toward reaching the learner's highest potential.

We also created the following definitions:

An *academic coach* is a person assigned to facilitate learners achieving their fullest potential. Coaches work with learners by evaluating performance *via* review of objective assessments, assisting the learner to identify needs and create a plan to achieve these, and helping the learner to be accountable. Coaches help learners improve their own self-monitoring, while modeling the idea that coaching will likely benefit them throughout their career.

In the *coaching relationship*, a trusted bond is formed that proceeds longitudinally. The coaching relationship should be built on respect rather than on friendship and should include navigating both negative and positive reactions.

### Identifying constructs: steps for effective coaching in medical education

Cummings and Worley suggest that effective coaching involves six steps, which we have condensed into four (3). We used these steps to identify and develop constructs for clarifying the coaching relationship and process. These categories can eventually lead to metrics by which coaching can be assessed. The steps are:

1. *Establishing Principles of the Relationship* – Initial coaching meetings should establish goals and parameters of the relationship, as well as ethical considerations, including confidentiality and boundary issues. The success of the relationship is important for the outcomes associated with the process to be effective.
2. *Conducting an Assessment –* This step involves both personal and systemic assessments. Personal assessments involve guiding the learner through frameworks to foster discovering the students’ learning or interpersonal management style. Systemic assessments include examining assessments provided by the learner's program. Review of both types of assessments must lead to a feedback process to begin self-monitoring and encourage learners gaining reflective skills to help them set goals for their program.
3. *Developing and Implementing an Action Plan* – This step includes goal setting, determining new and revised actions that will lead to goal attainment, identifying learning opportunities that build knowledge and/or skills, or initiating actions that will demonstrate the learner's progress toward competence. The learner must reflect on what is working and what is not working, relate these to learning style, and see new possibilities for action. Action plans should include specific methods, timelines, and milestones that allow both the coach and the learner to monitor progress. The learner designs the plan; the coach holds the learner accountable.
4. *Assessing the Results of Action Plans and Revising Accordingly*. At appropriate intervals, the coach and learner should review and evaluate how the student is progressing according to the action plan and whether features of the plan should be revised.

### Development of a coaching assessment instrument

After considering both our definition of coaching applied to medical education and the steps identified by Cummings and Worley, we identified key features of coaching that should be measured. As presented in Table 1, these fall into three constructs: 1) the coaching *relationship* or the trust, boundaries, credibility, expectations, engagement, and interdependence that occur between the learner and the coach, 2) the *coaching process from the perspective of the coaches*, such as appreciative listening, stimulation of insight, ongoing self-assessment, goal setting, and discerning problems and resources to solve them; and 3) the *coaching process from the perspective of the learners*, such as appreciative listing, reflection and processing, developing humility, ongoing self-assessment, goal setting, perceptions of needs, and problem identification and resolution. Future research will involve exploratory principal component analysis on these items.

**Table 1:** Working constructs of effective coaching

Construct	Definitional concepts	Person of relevance
		
Relationship	Trust – belief that a coach or student is reliable, good, honest, and effective	Both learners and coaches
	Boundaries – limits that define acceptable behavior	
	Credibility – quality or power of inspiring belief	
	Expectations – feeling or belief about how successful someone or something will be	
	Engagement – emotional involvement or commitment	
	Interdependence – state of reliance on and being responsible to each other	
		
The coaching process: faculty abilities	Appreciative listening – listening behavior where a listener seeks information that helps meet his or her needs and goals	Coaches
	Stimulating insight using inquiry – questioning in ways that help students develop the ability to perceive clearly	
	Ongoing informed self-assessment/self-monitoring – process of looking at oneself to identify central traits important to one's identity and enhancement	
	Shared agenda	
	Goal setting – establishing specific, measurable, achievable, realistic, and time-targeted goals	
	Discerning problems and referring to resources	
		
The coaching process: learner abilities	Appreciative listening – listening behavior where a listener seeks information that helps meet his or her needs and goals	Students
	Reflection and processing – capacity to exercise introspection and willingness to learn more about themselves	
	Humility (humble acceptance) – act or posture of acceptance of one's defects	
	Ongoing informed self-assessment/self-monitoring – behaving in a manner that is highly responsive to social cues and situational contexts	
	Goal setting – establishing specific, measurable, achievable, realistic, and time-targeted goals	
	Perceptions of needs – process by which individuals select, organize, and interpret information regarding what is essential to them	
	Identify problem, receive clarity, and solve own problems	

### Development and implementation of a coaching program

With the above concepts in mind, we have created and implemented a coaching program for medical students. We hired a cadre of 31 coaches, each of whom worked with five assigned students from every year of our curriculum. Coaches are paid 0.1 FTE, which covers dedicated time for monthly student meetings as well as monthly faculty development on coaching. Coaches use individual meetings to help students set and achieve concrete goals. These goals, as well as academic performance and attainment of competencies, are tracked using an electronic portfolio specifically designed to support our curriculum's individualized approach to medical education. Coaches are assessed by their students using an instrument comprising the constructs listed above as well as objective structured coaching examinations. Coaches also assess learners using these constructs.

## Implications

As medical knowledge continues to expand, physicians must become skilled in identifying gaps in knowledge and skills and continually embark on cycles of self-improvement. Coaching is emerging as a potential approach to facilitate this process, and it represents a shift from traditional advising and mentoring. With these proposed constructs and definitions, further research should be conducted to examine how to measure the coaching relationship and process and its effects on learning outcomes, lifelong self-directed learning, and overall academic development at varying skill levels.
